# Individual and Population Level Resource Selection Patterns of Mountain Lions Preying on Mule Deer along an Urban-Wildland Gradient

**DOI:** 10.1371/journal.pone.0158006

**Published:** 2016-07-13

**Authors:** John F. Benson, Jeff A. Sikich, Seth P. D. Riley

**Affiliations:** 1 La Kretz Center for California Conservation Science, Institute of the Environment and Sustainability, University of California Los Angeles, Los Angeles, California, United States of America; 2 National Park Service, Santa Monica Mountains National Recreation Area, Thousand Oaks, California, United States of America; Università degli Studi di Napoli Federico II, ITALY

## Abstract

Understanding population and individual-level behavioral responses of large carnivores to human disturbance is important for conserving top predators in fragmented landscapes. However, previous research has not investigated resource selection at predation sites of mountain lions in highly urbanized areas. We quantified selection of natural and anthropogenic landscape features by mountain lions at sites where they consumed their primary prey, mule deer (*Odocoileus hemionus*), in and adjacent to urban, suburban, and rural areas in greater Los Angeles. We documented intersexual and individual-level variation in the environmental conditions present at mule deer feeding sites relative to their availability across home ranges. Males selected riparian woodlands and areas closer to water more than females, whereas females selected developed areas marginally more than males. Females fed on mule deer closer to developed areas and farther from riparian woodlands than expected based on the availability of these features across their home ranges. We suggest that mortality risk for females and their offspring associated with encounters with males may have influenced the different resource selection patterns between sexes. Males appeared to select mule deer feeding sites mainly in response to natural landscape features, while females may have made kills closer to developed areas in part because these are alternative sites where deer are abundant. Individual mountain lions of both sexes selected developed areas more strongly within home ranges where development occurred less frequently. Thus, areas near development may represent a trade-off for mountain lions such that they may benefit from foraging near development because of abundant prey, but as the landscape becomes highly urbanized these benefits may be outweighed by human disturbance.

## Introduction

Identifying environmental conditions associated with specific activities that influence fitness of animals, such as foraging, is a fundamental pursuit in ecology and conservation biology [1, 2]. Understanding extrinsic influences on the acquisition of food resources is especially critical for the management of small populations in human-altered landscapes because suitable habitat may be limited, and fluctuations in demography due to environmental stochasticity can have serious, rapid consequences for extinction risk [3–5]. Thus, quantifying habitat and landscape features associated with successful foraging provides valuable information regarding the spatial ecology and habitat requirements of populations of conservation concern.

Large carnivores, such as mountain lions (*Puma concolor*), are sensitive to habitat fragmentation because they occupy large home ranges within which they must acquire sufficient large prey [6]. However, mountain lion populations are able to persist within urbanized landscapes in some situations, especially where protected lands exist within or adjacent to developed areas [7–9]. In these situations, it becomes imperative to understand predator-prey interactions and how they may be influenced by development and other landscape features encountered by mountain lions while foraging for preferred prey along urban-wildland gradients. Despite considerable interest in maintaining populations of mountain lions in ecosystems altered by anthropogenic activities (e.g., [7, 10, 11]), there is little information about predator-prey interactions and resource selection of mountain lions at predation sites within and adjacent to highly urbanized landscapes.

Ecologists are increasingly recognizing the importance of considering individual-level variation in resource selection studies [12]. A common source of variation for mountain lions are sexual differences, as males and females often use space and select resources differently [13–14]. However, most studies of resource selection specifically at foraging sites of mountain lions have not investigated sex-specific patterns [15–18], but see [14]. Another common source of individual variation in resource selection stems from spatial variation in the distribution and abundance of resources. For instance, selection of a resource may vary as a function of its availability on the landscape, often referred to as a functional response in resource selection [19]. Several studies have found that large carnivores exhibit functional responses to anthropogenic landscape features or activity, which may represent trade-offs between mortality risk and foraging success (e.g., [13, 20]). Knopff et al. [20] suggested that mountain lions in Alberta, Canada benefited from human-altered landscapes primarily through prey acquisition and found that mountain lions selected some anthropogenic features more strongly in areas where these features were more prevalent. Recently, Smith et al. [21] found that male and female mountain lions responded differently to increasing development in terms of kill rates and handling time of prey. Thus, both intrinsic (sex) and extrinsic (resource availability) sources of individual-level behavioral variation may influence mountain lion-prey interactions and resource selection in human-altered landscapes. Investigating mountain lion predation in more highly urbanized landscapes than previous studies, such as in areas within and adjacent to major metropolitan areas, may identify novel behavioral responses of these top predators to human disturbance.

We quantified selection of natural and anthropogenic landscape features at sites where mountain lions consumed mule deer (*Odocoileus hemionus*) across home ranges in and adjacent to the city of Los Angeles. Specifically, we addressed several questions. 1) What natural and anthropogenic landscape features were selected and avoided by mountain lions at foraging sites? 2) Were there differences in resource selection patterns between males and females? 3) Was there substantial additional individual-level variation in the responses of mountain lions to anthropogenic land-uses due to spatial variation in the level of human disturbance? Our results are important because they provide novel information about environmental conditions that facilitate successful predation by mountain lions in a highly urbanized landscape. This information will be valuable for managers attempting to conserve healthy populations of mountain lions, and naturally-functioning ecosystems, along urban-wildland gradients surrounding metropolitan areas.

## Methods

### Ethics statement

Animal capture and handling protocols were approved by the National Park Service Institutional Animal Care and Use Committee. We conducted research according to the conditions of animal capture and handling protocol PWR_SAMO_Riley_Mt.Lion_2014.A3. Mountain lions are not an endangered species in California. However, they are a specially protected species that cannot be legally killed in California except under specific circumstances with a state approved permit.

### Study area

**Fig 1:**
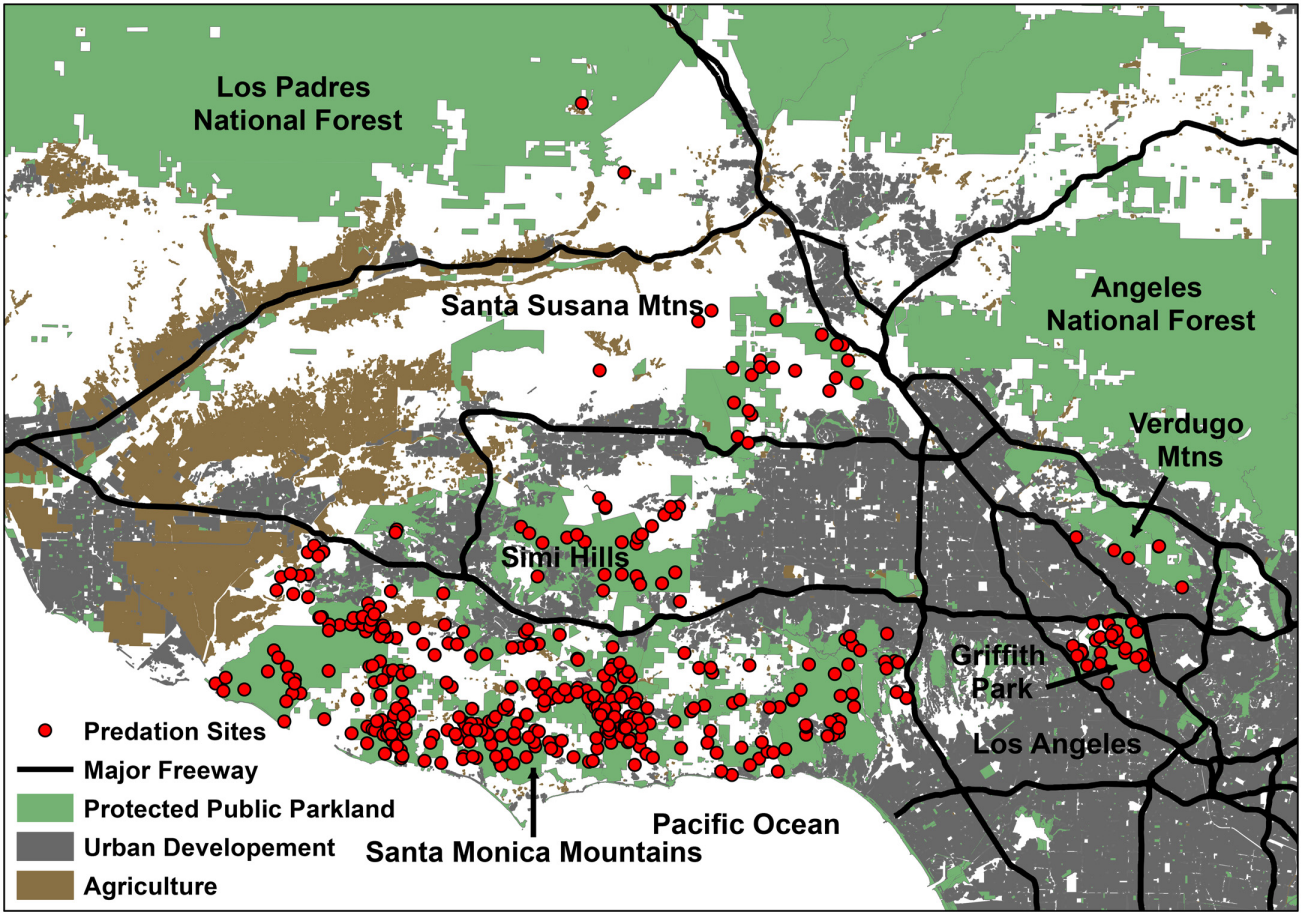
Greater Los Angeles area in southern California where we studied mountain lion predation on mule deer. Shown are sites where mountain lions fed on mule deer (Predation Sites), major freeways, protected parklands, areas of urban development, and agricultural areas.

We conducted research in and adjacent to the city of Los Angeles in Los Angeles and Ventura Counties, California (Fig 1). The study was focused on the Santa Monica Mountains National Recreation Area (SMMNRA; 34°05’N, 118°46’W), a unit of the National Park Service, and surrounding areas. Most research was carried out on public lands managed by the National Park Service who granted us access to the properties. When access was needed to private land this was obtained from private landowners in advance. The SMMNRA encompassed 600 km^2^ and included an assemblage of federal, state, and privately-owned lands largely in the Santa Monica Mountains. The Santa Monica Mountains were bordered by the Pacific Ocean to the south; by US 101, an 8–10 lane freeway, and various urban and suburban communities to the north; by the highly urbanized Los Angeles basin to the east; and by agricultural and developed areas in Ventura County to the west. Additionally, we studied mountain lions in areas north and east of the Santa Monica Mountains in the Simi Hills, the Santa Susana Mountains, Griffith Park, and the Verdugo Mountains (Fig 1). Griffith Park was a municipal park lying within the city of Los Angeles in the western portion of the Santa Monica Mountain range (Fig 1). The Verdugo Mountains were a small, rugged mountain range spanning several cities adjacent to Los Angeles including Glendale and Burbank (Fig 1). All patches of natural habitat in the study area were bordered by major freeways, urbanization, or agricultural development. The study area was characterized by a Mediterranean climate, with cool, wet winters and hot, dry summers. There were multiple land uses throughout the area including federal, state, and local parklands, urban and suburban areas with commercial and residential (both high and low density) development, and agricultural areas (Fig 1). Natural vegetation consisted of mixed chaparral, coastal sage scrub, oak woodlands and savannas, riparian woodlands, and non-native annual grasslands. Bobcats (*Lynx rufus*) and coyotes (*Canis latrans*) occurred throughout most of the study area and the only wild, large ungulates were mule deer.

### Capture and telemetry

We captured mountain lions using Aldrich foot-snares or cable-restraints, baited cage-traps, or by treeing them with trained hounds during 2002–2015. We immobilized mountain lions with ketamine hydrochloride combined with either xylazine hydrochloride or medetomidine hydrochloride administered intramuscularly. Animals were monitored for the duration of the time they were immobilized. We deployed global positioning system (GPS) collars (Followit AB, Simplex and Tellus models, Stockholm, Sweden; North Star Science and Technology LLC, Globalstar Tracker model, King George, Virginia, USA; or Vectronic Aerospace, GPS Plus model, Berlin, Germany) equipped with VHF beacons on adults. Fix schedules of GPS collars varied but we programmed most collars to obtain 1–2 day locations and 5–7 night locations per 24/hour period. Additional details of our capture, handling, and tracking of mountain lions are available elsewhere [8].

### Feeding site investigations

We found carcasses of prey eaten by mountain lions by visiting clusters of GPS or VHF telemetry locations (*n* = 520) and searching the area for prey remains. When we found prey remains (*n* = 473) we identified the species, sex, and age of the prey when possible. We obtained coordinates at the prey remains with a handheld GPS unit. Previous research has indicated that clusters of mountain lion locations with locations from >1 night had a high probability of containing a kill, and that increasing numbers and the proportion of night locations in GPS clusters were strong predictors of sites where mountain lions fed on kills [18, 21, 22, 23]. Our protocol for finding prey remains was to visit clusters of telemetry locations that contained ≥ 2 night (sunset-sunrise) locations within ≤ 50 meters of each other and spanning periods ≥ 24 hours. We found prey remains at 91% of the clusters we visited. We reduced the likelihood of bias in the location of these carcasses with respect to landscape features because we visited clusters of locations regardless of the topography or their proximity to habitat features or roads.

A previous study of mountain lions in southern California found that large prey are generally dragged 0–80 m from the kill site [24]. Thus, we recognize that most of the locations where we found carcasses were probably not the exact locations where the deer were killed. However, our distance-based analysis was robust to location error [25] which ensured that our results should provide reliable inferences regarding areas where deer were killed and consumed. Nonetheless, we refer to the locations where we found carcasses as feeding sites to reflect that the deer were not necessarily killed exactly at these sites.

### Resource use and availability

We investigated resource selection with an approach similar to Johnson’s (1980) 3^rd^ order of selection by comparing locations used by mountain lions at feeding sites to those available within their annual home ranges. The use-available design for resource selection models estimates the relative probability of use of resource variables (i.e. relative to their availability). For the used locations, we identified 30 m pixels (30 x 30m) on the landscape within which we found carcasses of mule deer preyed upon by mountain lions. We also occasionally found carcasses of smaller prey species (*n* = 54, 11% of kill sites) but we chose to focus our analysis on deer because they comprised the majority of carcasses and because mountain lions rely on large ungulates to survive and reproduce [13]. We had small sample sizes for smaller prey species (*n* ≤ 33 for each individual prey species smaller than deer). Additionally, given that mountain lions likely spent considerably less time at sites where smaller prey items were consumed, we assumed we missed more of the smaller prey.

To estimate availability, we estimated adaptive local convex hull home ranges in R version 2.15.1 with the package ‘adehabitat’ using GPS telemetry data for each mountain lion included in our analysis. We set the ‘a’ parameter as the maximum distance between any 2 points in each dataset [26]. In total, we estimated 49 home ranges for 26 mountain lions with telemetry data (mean number of locations = 1814, range = 166–5507) collected across 1–12 months (mean number of monitoring days = 247, range = 30–365). In most (90%) cases we estimated home ranges with data specific to a single calendar year that matched the year of the feeding sites identified for that mountain lion. In the remaining cases (10%) we combined continuous data from 2 consecutive calendar years (if < 40 days of data were available within a single year) to provide a better estimate of space use for that individual. We used calendar year as our temporal unit rather than season because seasonal variation in climate and conditions was relatively subtle in our southern California study area.

We systematically sampled 30 m pixels separated by 150 m throughout each annual home range resulting in 44 pixels/ km^2^ to estimate resources available to each mountain lion [27]. We calculated distances to habitat types, land use designations, and roads from the centroid of all 30 m pixels used by (feeding sites) and available to (systematic locations across each individual’s home range) mountain lions while preying on deer. Additionally, we classified the slope and elevation values associated with used and available pixels. We modified 2 existing habitat/vegetation layers (Santa Monica Mountains National Recreation Area Vegetation Layer, 2007 and the CALVEG—South Coast Layer [CALVEG, USDA-Forest Service, Pacific Southwest Region, 2013]) by combining similar habitat types to produce a layer with 6 broad habitat classes: chaparral, coastal sage scrub, prairie/meadow, upland woodland, riparian woodland, and water (Table 1). For areas where natural habitat was developed or otherwise altered for anthropogenic activities, we generalized a digital land-use map for 2 classes of anthropogenic land use: developed areas (3% of mountain lion home ranges) and altered-open areas (9% of home ranges). Developed areas included commercial and residential areas with ≥1 house/acre. Altered-open areas were modified by humans to a lesser extent than developed areas and included golf courses, schools, landscaped areas such as city parks, low-density residential areas (<1 house/acre), cemeteries, horse ranches, and other moderately developed areas. We calculated distances from the centroid of all used and available pixels to the closest pixel of the 6 habitat classes and 2 land-use classes using the ‘Euclidean Distance’ tool in the Spatial Analyst toolbox in ArcGIS 10.2.2 (ESRI, Redlands, CA, USA) using Geographic Information System (GIS) methods described by Benson [27]. We also estimated distances from used and available locations to 3 classes of paved roads, as well as a single class of unpaved roads and trails (hereafter referred to as trails). Additionally, we estimated slope and elevation from digital elevation models (DEM) in ArcGIS (Table 1). DEM data were estimated at 9.5 m resolution, but we averaged these data across 30 m used and available pixels for our analyses. These distance and classification based values allowed us to compare resources used by and available to lions while preying on deer.

**Table 1:** Resource variables included in resource selection function models for feeding sites used by mountain lions preying on mule deer in southern California, 2002–2015.

Resource variable	Type	Range (units)	Description
Slope	Continuous	0–70 (%)	Inclination of terrain from horizontal
Elevation	Continuous	0–2432 (m)	Vertical distance above sea level
Chaparral	Continuous	0–3027 (m)	Dense, drought and fire-adapted evergreen shrubs
Riparian woodland	Continuous	0–8645 (m)	Diverse riparian forest and shrubs along canyon bottoms and drainages
Upland woodland	Continuous	0–8008 (m)	Oak, walnut, and other woodlands on slopes, shaded ravines, and canyons
Prairie/Meadow	Continuous	0–5396 (m)	Open grasslands
Water	Continuous	0–9817 (m)	Lakes, ponds, creeks, reservoirs
Trail	Continuous	0–7878 (m)	Unpaved roads and trails
Developed areas	Continuous	0–11545 (m)	Residential and commercial development
Altered-open areas	Continuous	0–5858 (m)	Areas modified by humans but less so than developed areas
Male	Categorical	0–1	Female (0) or male (1)

Distance-based variables are effective for assessing habitat selection [28] and using continuous, distance-based variables for habitat classes (rather than categorical variables) also eliminated the need to base inference on subjectively chosen reference categories in our regression models [29]. Distance-based approaches for habitat selection analysis are also robust to error in location data [26] and mitigate GIS error. Thus, even though mountain lions likely dragged carcasses short distances from the actual kill sites, our distance-based analysis should capture selection of areas where lions killed and consumed deer. Distance-based analyses are especially effective for assessing resource-selection at individual sites on the landscape, such as feeding sites, because the proximity to specific resource variables (e.g., water or developed areas) is quantified even if the sites of interest rarely or never actually fell within the habitat types or land-use types being considered.

### Resource-selection models

We modeled resource selection at feeding sites with generalized linear mixed models (GLMMs) implemented in the R (version 3.1.1) package ‘lme4’with a binary (0 = available, 1 = used) response variable. We included random intercepts for individual and year in each model, with year nested in individual. Including random intercepts for individuals mitigated the effects of the unbalanced feeding site data across individuals (range 1–77) and the lack of independence between used locations from the same individual [30]. The random intercept of year accounted for correlation between sites used within a given year by a given individual and paired the year-specific used and available data appropriately within our models. We did not include the paved road classes in our resource selection models because of 1) the infrequency of major highways (primary roads) within mountain lion home ranges, and 2) correlation of intermediate-sized paved roads (secondary roads) and smaller paved roads (tertiary roads) with developed (*r* = 0.53) and altered-open (*r* = 0.57) areas, respectively. Secondary and tertiary roads were also correlated with each other (*r* = 0.55). Finally, we excluded the coastal sage-scrub habitat class because it was correlated (*r* = 0.63) with elevation. We were more interested in investigating and accounting for the general influence of elevation on selection of feeding sites than the influence of the specific habitat-type of coastal sage-scrub. Correlation between other predictor variables was modest or low (*r* < 0.44) so we included all remaining variables in our global model (Table 1). Prior to modeling, we rescaled values for all continuous variables by subtracting their mean and dividing by 2 standard deviations following Gelman [31].

The terms “selection” and “preference” have sometimes been used synonymously or inconsistently in the resource selection literature [29, 32]. To avoid confusion, we use the term selection throughout to indicate 1) that used locations (feeding sites) were significantly closer to distance-based resource variables (habitat types, land-use types, trails) than were available locations, or 2) that values of classification-based resource variables (elevation and slope) were significantly greater at used locations relative to available locations. Specifically, we inferred selection or avoidance of resource variables when 95% or 90% confidence intervals of fixed-effect beta coefficients did not overlap 0.

We investigated potential sex-specific patterns in resource selection at feeding sites by including a dummy-coded ‘male’ variable (0 = female, 1 = male) and fitting interactions between ‘male’ and each resource variable. We compared the fit of the null model, the global model with all resource variables, a model with interactions between ‘male’ and each resource variable, and a reduced interactions model retaining only interactions that indicated or approached significance (i.e. when 85% confidence interval did not overlap 0) using Akaike’s Information Criteria (AIC; [33]). This allowed us to evaluate support for models of varying complexity by calculating differences (Δ) in AIC values (lower values indicate better fit). We concluded there was strong empirical support for more complex models if ΔAIC > 5. We investigated the significance and marginal significance of fixed effects and interactions in strongly supported models with 95% and 90% confidence intervals, respectively.

We tested the predictive ability of our models using k-fold cross validation implemented in ‘lme4’ as described by Boyce et al. [34]. Specifically, we used 80% of the data (training data) to build a model that was then used to predict the relative probability of use of the remaining 20% (test data). This procedure was repeated 5 times until all data had been used as both training and test data. We ran Spearman rank correlations to assess relationships between the frequency of cross-validated feeding site locations and 10 probability bins of equal size representing the range of predicted values. A model with good predictive ability is expected to show a strong correlation with higher numbers of locations falling into higher probability bins [34].

### Functional responses to human disturbance

We also explored additional individual variation in selection of areas modified by humans. Specifically, we hypothesized that distance of mule deer feeding sites to developed or altered-open areas might vary as a function of the availability of these areas, consistent with a functional response in resource selection as defined by Mysterud and Ims [19]. To test these hypotheses, we constructed resource-selection proportions by dividing the mean distance at used locations by the sum of the mean used and available locations (mean used distance / [mean used + mean available]). Proportions of 0.5 represented no difference between used and available, proportions < 0.5 indicated mountain lions were closer to the feature than expected, and proportions > 0.5 indicated mountain lions were farther from the feature than expected. We averaged selection proportions and availability values across years for individuals with feeding sites documented in multiple years. We only constructed these proportions for mountain lions for which we documented ≥ 5 feeding sites (*n* = 19). We explored potential functional responses by modeling these resource-selection proportions (response variable) as a function of distance-based resource availability of the same resource (predictor variable, i.e. mean distance to the feature of interest across home ranges) using beta regression models with a ‘probit’ link which is appropriate for conducting regression on a proportional response variable. We fit 2 separate models for developed and altered-open areas using generalized additive models (GAMs) in the R package ‘mgcv’ version 1.8–0. We specified predictor variables as non-parametric smooth functions (splines) in the GAMs to allow for the possibility of non-linear relationships, which are often observed with functional responses [12]. However, if the relationship between the response and predictor variables was better modeled as linear (i.e. estimated degrees of freedom [edf] = 1) then predictor terms were included as parametric fixed effects [35]. Thus, because semi-parametric GAMs do not assume a linear response, they allowed us to evaluate whether selection of anthropogenic resources by individual mountain lions varied as a linear or non-linear function of availability. Small samples precluded testing for differences in functional responses between sexes. However, we visually inspected plots of the raw data to ensure that pooling sexes was not disguising obvious differences and that these relationships were qualitatively similar between sexes.

## Results

### Feeding sites

We included the locations of 420 mule deer killed by 26 mountain lions (16 males, 10 females) in our analysis. We found 229 mule deer killed by mountain lions in chaparral (55%), 91 in coastal sage-scrub (22%), 52 in upland forest (12%), 14 in prairie-meadow (3%), 8 in riparian-woodland (2%), 8 in disturbed areas (2%), 8 in altered-open areas (2%), 5 in exotic vegetation (1%), 2 in rocky outcrops (<1%), 2 in developed areas (<1%), and 1 in water (<1%). In reality, the 1 kill classified as being in water was located close to (rather than in) water. This kill was < 5m from the edge of a water patch in our habitat layer and was classified as water because it fell within the boundary of a pixel classified as water.

### Population-level and sex-specific resource selection

**Fig 2:**
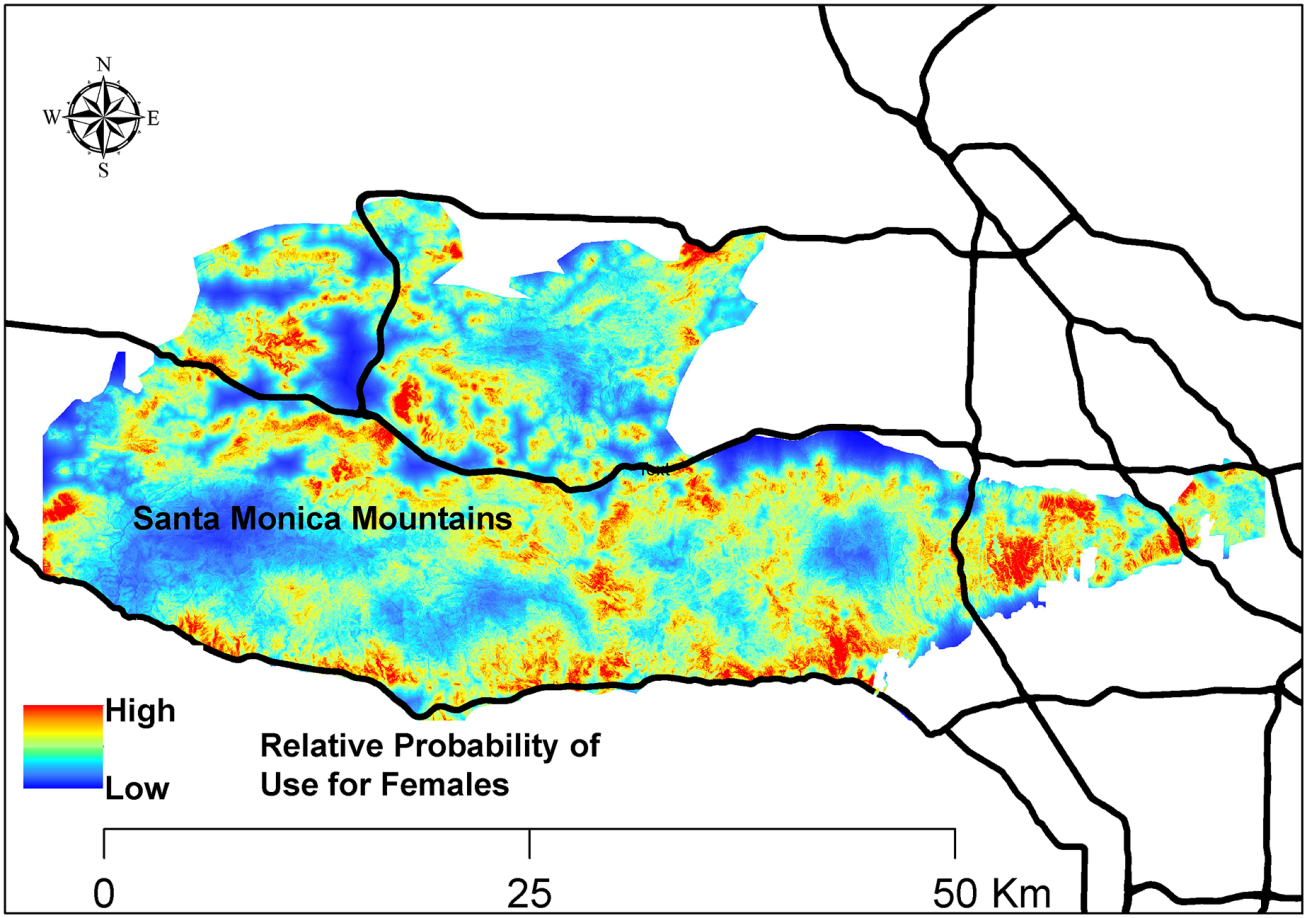
Relative probability of use of mule deer feeding sites by female mountain lions in the Santa Monica Mountains and Simi Hills, southern California, 2002–2015. Relative probability of use predicted by generalized linear mixed model of resource selection.

**Fig 3:**
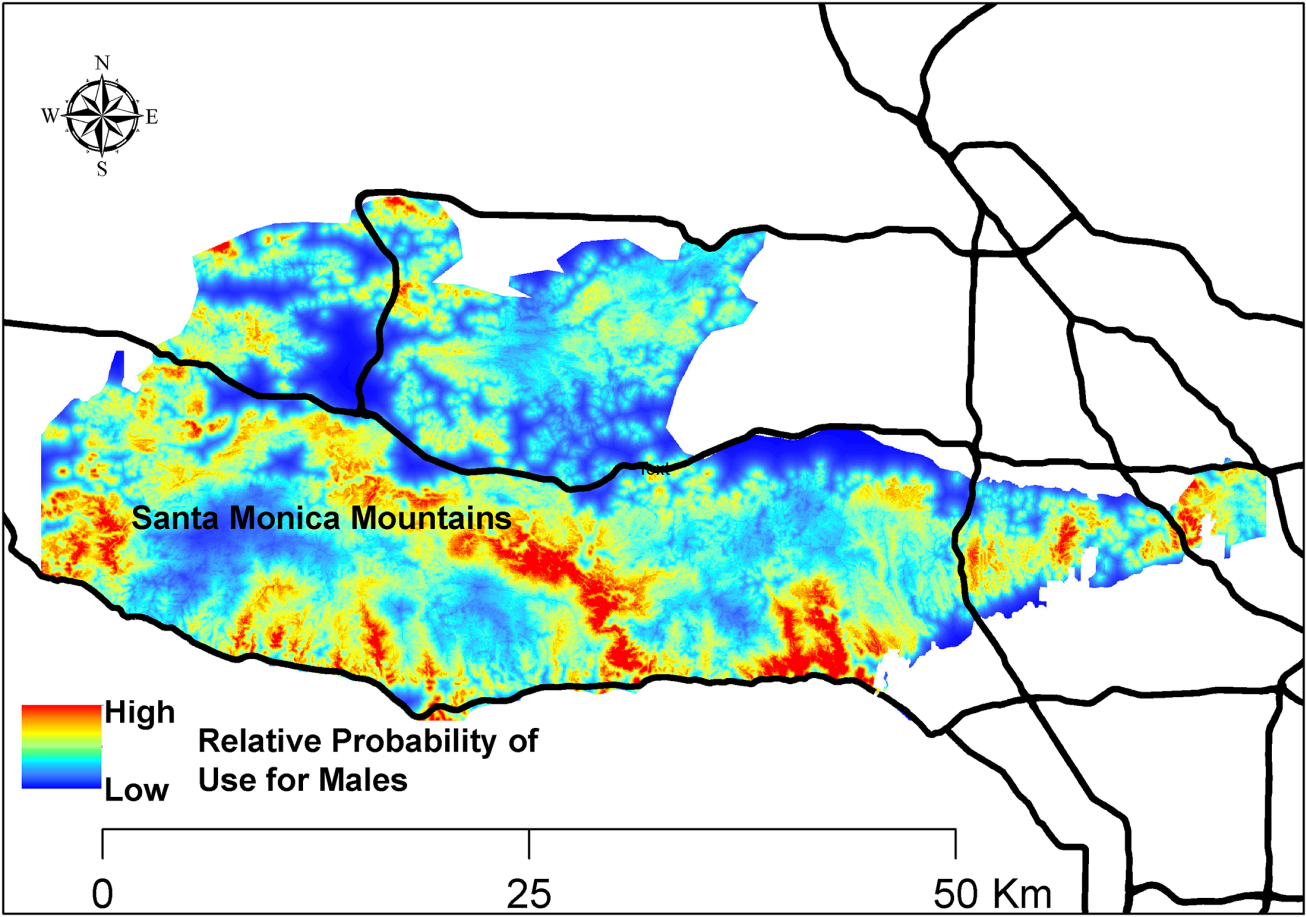
Relative probability of use of mule deer feeding sites by male mountain lions selection in the Santa Monica Mountains and Simi Hills, southern California, 2002–2015. Relative probability of use predicted by generalized linear mixed model of resource selection.

Model fit for models with resource variables was considerably better than for the null model, indicating that the resource variables provided substantial information regarding resource selection at mule deer feeding sites (Table 2). There was also support for considering differences in resource selection at feeding sites between male and female mountain lions (Table 2). Model fit was best with the reduced interactions model which accounted for sex-specific differences in selection of riparian woodland, water, and developed areas (Table 2). Males selected riparian woodland and water more than females, whereas females selected developed areas marginally more than males (Table 3, Figs 2 and 3). Main effects for the resource variables fit with interactions indicated that females significantly selected developed areas, whereas they avoided riparian woodlands (Table 3). Females did not select or avoid water (Table 3). There were not significant sex-specific differences for other resource variables. Mountain lions avoided higher elevations, whereas they selected steeper slopes, chaparral, and upland forests at mule deer feeding sites (Table 3). Mountain lions also marginally selected trails at mule deer feeding sites (Table 3). Sex-specific maps of the relative probability of use of mule deer feeding sites by males and females are shown in Figs 2 and 3. The best resource-selection model, with 3 sex-specific interactions, had good predictive ability as the frequency of cross-validated locations within probability bins were highly correlated with bin ranks (*r*_*s*_ = 0.85).

**Table 2:** Comparison of model fit between models of varying complexity. Shown are Akaike’s Information Criteria (AIC) and differences between best model and competing models (ΔAIC).

Model	AIC	ΔAIC
3 resource × sex interactions*	5927.7	0
All resource variables, no interactions	5934.4	6.7
All possible resource × sex interactions	5937.2	9.5
Null model	6014.1	86.4

* Riparian woodland × male, water × male, developed areas × male

**Table 3:** Results of mixed-effect resource selection models for mountain lions at mule deer feeding sites in and adjacent to Los Angeles in southern California, 2002–2015. Shown are β coefficients and 95% and 90% confidence intervals. Significant and marginally significant fixed effects, based on 95% and 90% confidence intervals, respectively, shown in bold. Note that for classification-based variables (elevation and slope) positive β indicate selection, negative β indicate avoidance. All other variables are distance-based, so negative β indicate selection, positive β indicate avoidance. Also shown are the mean values at mule deer feeding sites used by mountain lions.

	β	95% LCL	95% UCL	90% LCL	90% UCL	Mean used value
Intercept	-6.84	-7.71	-5.98	-7.57	-6.10	——
Male	-0.76	-1.88	0.36	-1.70	0.18	——
Elevation	**-0.93**	**-1.28**	**-0.58**	**-1.22**	**-0.63**	372 (m)
Slope	**0.23**	**0.03**	**0.44**	**0.06**	**0.41**	24 (%)
Water	-0.04	-0.45	0.38	-0.39	0.30	2523 (m)
Riparian woodland	**0.74**	**0.09**	**1.39**	**0.19**	**1.28**	808 (m)
Chaparral	**-0.97**	**-1.46**	**-0.37**	**-1.43**	**-0.51**	54 (m)
Prairie-meadow	-0.02	-0.26	0.23	-0.22	0.19	578 (m)
Upland woodland	**-0.58**	**-0.97**	**-0.18**	**-0.91**	**-0.4**	273 (m)
Altered open	0.06	-0.23	0.34	-0.18	0.30	651 (m)
Developed	**-1.05**	**-1.61**	**-0.48**	**-1.52**	**-0.58**	1325 (m)
Trails	**-0.43**	-0.80	0.06	**-0.74**	**-0.12**	459 (m)
Riparian woodland × male	**-1.06**	**-1.84**	**-0.28**	**-1.71**	**-0.40**	**——**
Water × male	**-0.62**	**-1.15**	**-0.09**	**-1.06**	**-0.18**	**——**
Developed × male	**0.66**	-0.04	1.37	**0.07**	**1.26**	**——**

### Functional response modeling

**Fig 4:**
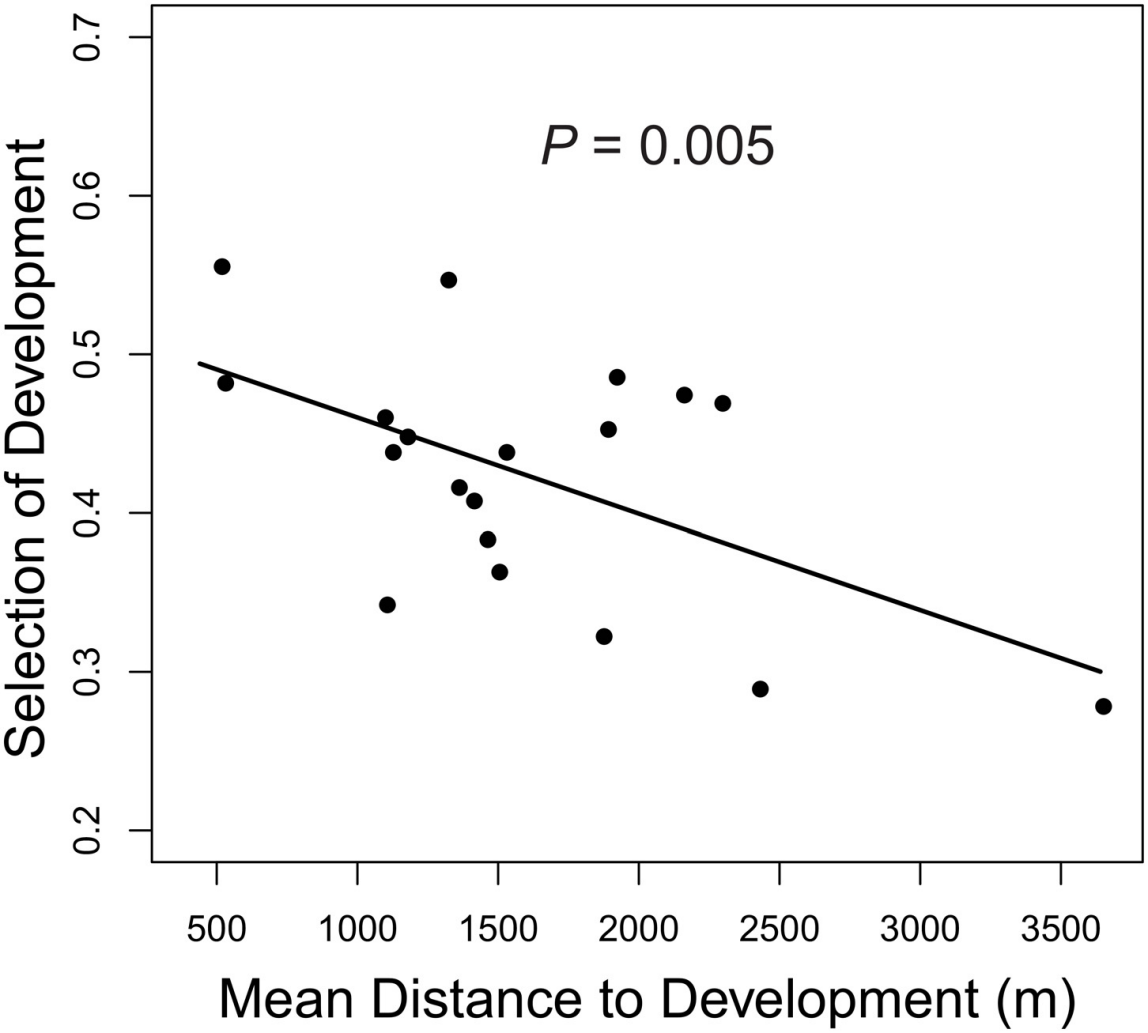
Functional response by mountain lions feeding on mule deer relative to development. Functional response is exhibited by the linear relationship between a selection proportion for developed areas (used distance/ used + available distance; y axis) and the mean distance to developed areas across each mountain lions home range (x axis). Selection proportions < 0.5 indicates mountain lions were closer than expected, whereas proportions > 0.5 indicate mountain lions were farther than expected from the resource relative to availability.

Mountain lions exhibited a functional response to developed areas at feeding sites as there was a significant, negative relationship between selection of developed areas and mean distance to developed areas within individual home ranges (χ^2^ = 7.89, *P* = 0.005, Deviance explained = 32.7%, *n* = 19) which did not differ from a linear trend (edf = 1.0; Fig 4). In general, mountain lions selected developed areas at mule deer feeding sites more within home ranges where developed areas were less common (Fig 4). Mountain lions did not select altered-open areas as a function of their availability (χ^2^ = 1.0, *P* = 0.541, edf = 1.6, *n* = 19).

## Discussion

Our results indicate that multiple environmental variables influence the location of mountain lion-mule deer predation events in the fragmented landscape along the urban-wildland gradient in the greater Los Angeles metropolitan area. Unfortunately no studies of mule deer have been conducted within our study area, so there is little information about mule deer abundance and distribution relative to habitat types and other environmental variables. Nonetheless, our results clearly reflect areas where mule deer were present, as well as where they were vulnerable to predation by mountain lions. Given the importance of obtaining sufficient large ungulate prey for maintaining healthy populations of mountain lions [13], understanding where mountain lions are able to kill mule deer is a valuable first-step in understanding the spatial ecology and resource selection of mountain lions in and adjacent to the Santa Monica Mountains.

Females selected mule deer feeding sites closer to developed areas at the population-level, and fed on deer marginally closer to developed areas than males, relative to availability. We point out that this selection was in a distance-based context and that no feeding sites for females were actually located within developed areas (mean distance to developed areas = 1316 m, range = 95–5165 m, *n* = 186 sites from 10 females). Thus, female mountain lions fed on mule deer closer to developed areas than expected, rather than within developed areas. We did find 2 feeding sites for males within developed areas (mean = 1304 m, range 0–4773, *n* = 234 sites from 16 males). Wilmers et al. [14] also documented female mountain lions selecting developed areas more than males (while traveling) and suggested that deer may be more abundant in developed areas in central California. Indeed, deer are often attracted to disturbed areas and cultivated vegetation [36]. In southern California, mule deer might also be abundant near developed areas due to the paucity of natural water sources, as residences provide water from sprinklers, swimming pools, and other sources. The extreme drought in southern California from 2012–2015 may have made these anthropogenic water sources, and the relatively lush vegetation they support, particularly important to deer.

However, there was additional individual variation in the selection of developed areas at the individual-level that was consistent with a functional response in resource selection. Mountain lions with home ranges with greater availability of developed areas selected developed areas less than those in more remote areas. This finding suggests that some developed areas can be beneficial to mountain lions and further supports previous contentions that anthropogenic disturbance is associated with greater prey availability [14, 20]. However, developed areas may also represent increased mortality risk for mountain lions [37]. Thus, there may be a trade-off for mountain lions associated with development as the potential negative effects of human disturbance appeared to outweigh the benefits at higher levels of urbanization. The developed areas in the highly urbanized portions of the study area, such as the densely populated neighborhoods surrounding Griffith Park and the Verdugo Mountains were characterized by higher levels of human activity and tended to be larger than developed areas in remote areas such as the Santa Monica Mountains (see Fig 1). Thus, both the size (as seen in Fig 1) and availability (quantified in our functional response analysis) of developed areas may influence where mountain lions kill mule deer. Killing and consuming deer farther from developed areas in more populated areas appears to be one way that mountain lions minimize interactions with humans in southern California.

The functional response we documented was contrary to the response exhibited by mountain lions in Alberta, where individuals decreased their avoidance of oil and gas pipelines and well sites as these features became more prevalent on the landscape [20]. There are several potential explanations for this apparent discrepancy. First, human populations and activity were considerably greater in the Los Angeles area (18.5 million people) than in west-central Alberta where populations in communities and rural areas ranged from 515–7231 people [20]. Thus, in rural areas that are relatively sparsely populated, mountain lions may select anthropogenic features more as these features become more abundant to compensate for reductions in natural habitat and to exploit potentially greater prey availability in these areas, as suggested by Knopff et al. [20]. When human presence and development reaches higher levels, as in southern California, mountain lions may stop selecting these features or even avoid them. Second, although deer may be attracted to lower and intermediate levels of disturbance and development [14, 36], they may be less common in highly developed areas in southern California making these areas less attractive to mountain lions.

Mountain lions in general resource selection studies in Washington and central California did not exhibit functional responses to development [14, 38]. Although these studies were conducted in human-altered landscapes, the mountain lions they studied almost certainly did not encounter human population densities as high as those found within and adjacent to Los Angeles, the second largest metropolitan area in the United States. That our results differ from areas where human population sizes are lower highlights the important and unique nature of our study area where mountain lions can be studied across a steep gradient of human disturbance that spans areas from within the city of Los Angeles, to relatively remote areas of protected lands within the Santa Monica Mountains. Understanding the range of behavioral responses of mountain lions to varying levels of urbanization and human disturbance is critically important if we are to maintain healthy populations of top predators, and relatively intact ecosystems, as development, urbanization, and human populations continue to increase.

In the strongest intersexual difference documented by our analyses, males showed strong selection of riparian woodlands, whereas females avoided these areas. Riparian woodlands might be attractive to male mountain lions because they are visited by mule deer taking advantage of the relative abundance of lush vegetation in these habitat patches in the dry Mediterranean landscape. Indeed, previous studies have shown that mule deer select riparian habitat types and that they are distributed in close proximity to water and succulent vegetation [39–41]. Additionally, riparian woodlands may be used by males because they represent corridors that facilitate travel across their large home ranges as these woodlands offer vegetative cover and terrain that is likely easier to traverse than the steep ridges and rugged canyons that are common throughout the study area. Although riparian woodlands may also be attractive to females, we suspect females may have avoided them to minimize encounters with aggressive males. The leading cause of death for mountain lions in our study area is intraspecific aggression, as males often kill other mountain lions including females and their offspring [8]. Confrontations over prey carcasses are thought to be a major reason why male mountain lions kill conspecifics [13]. Interestingly, an earlier study of general habitat selection by mountain lions in the Santa Ana Mountains adjacent to Los Angeles showed that riparian woodlands were selected above all other habitats by a sample of mountain lions that was mostly females [42]. This apparent discrepancy may be explained by the fact that we studied feeding sites specifically, or because intraspecific aggression does not appear to be a major source of mortality for mountain lions in the Santa Ana Mountains [43]. Thus, the higher intraspecific mortality risk in our study area may lead to stronger intersexual differences in resource selection.

The difference in the use of water by males and females may also reflect a trade-off made by females between selecting feeding sites where prey are abundant and vulnerable, and avoiding encounters with males. Standing water in ponds and streams is relatively rare in our study area and these water sources, and the vegetation they support, likely attract abundant mule deer. Indeed, proximity to water sources was a strong predictor of mule deer fawn and doe distribution in San Diego County [40]. However, anthropogenic sources of water (e.g., pools and sprinklers) are available in our study area and also may attract deer. It is possible that males prey on deer closer to natural water sources, whereas females prey on deer closer to developed areas which represent alternative areas where water is available and deer congregate. Given that mountain lion hunting is illegal in California, and that human activity in many developed areas would be relatively low at night when mountain lions are killing and eating deer, this trade-off could be beneficial from a fitness standpoint. However, Smith et al. [21] recently showed that females left mule deer kills sooner and compensated with higher kill rates in areas of greater development in central California. This suggests that there could be energetic costs for females preying on mule deer near developed areas. Although avoiding areas of natural habitat where deer are abundant and vulnerable may reduce intraspecific mortality risk, increased energetic expenditures associated with foraging near developed areas could potentially negatively influence fitness of females by lowering reproductive output or offspring survival.

Finally, previous studies have found that mountain lions select habitats with dense understory vegetation and noted the importance of adequate stalking cover to allow for successful hunting [14, 25, 38, 42]. Our results support these findings as chaparral, a habitat-type characterized by extremely dense shrubs, was selected by mountain lions at mule deer feeding sites and males strongly selected riparian woodlands which would also have dense stalking cover. Correlation between elevation and distance from coastal sage scrub habitat prevented us from completely separating avoidance of higher elevations and selection for coastal sage scrub. Exploratory analyses suggested both variables were important when included separately in different models, although the avoidance of higher elevation was considerably stronger than selection for coastal sage scrub. We chose to include elevation in our final model to avoid conducting regression with strongly correlated variables, but we do not suggest that coastal sage scrub is not potentially important foraging habitat for mountain lions in southern California. That 22% of the mule deer carcasses killed by mountain lions we documented were discovered in coastal sage scrub habitat highlights this point.

Our results and maps of relative probability of use of mule deer feeding sites should be incorporated into land management decisions in the Santa Monica Mountains National Recreation Area, as well as in other state, federal, city, and private parks and protected areas across the greater Los Angeles area to conserve mountain lions and naturally-functioning predator-prey dynamics. Our finding that mountain lions feed on their primary prey farther from development relative to availability as a function of increasing urbanization is broadly important because it extends understanding of how anthropogenic disturbance influences mountain lion resource selection at feeding sites with data in and around a major metropolitan area. Our results suggest that continued development in areas used by mountain lions adjacent to Los Angeles and other metropolitan areas could reduce the quality of foraging habitat for mountain lions, as they fed on mule deer farther from development when there was greater availability of developed areas within their home ranges. This functional response also indicates that, at least with respect to their behavior while preying on mule deer, mountain lions in and adjacent to Los Angeles appear to exhibit behavior that should reduce encounters and potential conflicts with humans. Our current results should be linked with analyses of resource selection by mountain lions during other activities, such as traveling and denning, to provide a more comprehensive understanding of how mountain lions use the landscape along the urban-wildland gradient in southern California. Our current model, combined with additional resource-selection analyses, could be used to evaluate the potential influence of new development projects on the relative probability of use of these areas by mountain lions.
